# Intraoperative Cell-Saver Caused More Autologous Salvage Hemolysis in a Hereditary Spherocytosis Patient Than in a Normal Erythrocyte Patient

**DOI:** 10.3389/fphys.2022.926398

**Published:** 2022-06-30

**Authors:** Di Jin, Le Shen, Yuguang Huang

**Affiliations:** ^1^ Department of Anesthesiology, Peking Union Medical College Hospital, Beijing, China; ^2^ State Key Laboratory of Complex Severe and Rare Diseases (Peking Union Medical College Hospital), Beijing, China

**Keywords:** hereditary spherocytosis, autologous blood transfusion, hemolysis, Cell Saver, red blood cell, membrane defect

## Abstract

Hereditary spherocytosis is a common red blood cell disease caused by an inherited red blood cell membrane defect, leading to a spherical shape and propensity for hemolysis. There is a lack of reports on intraoperative autologous blood transfusion for hereditary spherocytosis patients. We hereby report our recent experience with using the Cell Saver^®^ system for intraoperative red blood cell salvage on a hereditary spherocytosis patient. There was a drastic increase in salvaged blood free-hemoglobin compared with the preoperative sample (82.6 mg/dl vs. 6.2 mg/dl) which indicated severe hemolysis. Although our patient recovered smoothly with a normal liver and renal function test and reported no adverse reaction during follow-up, it is noteworthy that severe hemolysis could happen during the cell salvage process for patients with hemolytic anemia, as there are similar reports on sickle cell anemia, beta-thalassemia intermedia, and paroxysmal nocturnal hemoglobinuria. Therefore, more clinical attention and thorough research should be drawn into this perspective, namely, hemolysis during the red blood cell salvage process for patients with hemolytic anemia.

## Introduction

Hereditary spherocytosis (HS) is a common red blood cell disease that is caused by inherited RBC membrane defects that cause the shape of red blood cells to become spherical and has a propensity for hemolysis (Tao et al., 2016). It is the most common cause of hemolytic anemia (Perrotta et al., 2008), but the severity of hemolysis varies widely; some patients may have normal hemoglobin (Hb) levels and are diagnosed when signs of chronic hemolysis (gallstone and splenomegaly, etc.) appear. The diagnosis of HS should be confirmed with tests including the erythrocyte osmotic fragility (EOF) test, acidified glycerol lysis test, or eosin-5′-maleimide binding test in patients without typical clinical manifestation or family history (Kalfa, 2021; Wu et al., 2021). Patients with HS often seek surgical treatment, as splenectomy is performed for patients with severe hemolysis, and cholecystectomy is performed for patients with symptomatic gallstones.

We presented a recent HS patient who underwent splenectomy and cholecystectomy, with another non-HS patient who underwent posterior correction of scoliosis as the control. They both received autologous blood transfusion via the same CS system, and the blood cell smear, osmotic fragility test, and free hemoglobin test were performed before and after the salvage process. Approval from the Institutional Review Board of Peking Union Medical College Hospital was obtained. Both patients gave written informed consent to participate in the study.

## Case Presentation

The HS patient is a 26-year-old female who was admitted for persistent jaundice and the recent onset of right upper quadrant pain. Previous medical history revealed persistent anemia for 10 years and elevated bilirubin. The lab test showed a positive EOF test (defined as a higher saline concentration to begin hemolysis, no less than a 0.08% difference), a negative Coombs test, and an elevated mean cell-free hemoglobin concentration. Spherocytes were observed on the peripheral blood smear, and the patient was diagnosed with HS. Her abdominal computed tomography (CT) scan showed an enlarged spleen and cholecystitis with multiple gallstones. She was scheduled for splenectomy and cholecystectomy. The control non-HS patient is a 12-year-old female with congenital scoliosis who was scheduled for posterior correction of scoliosis and internal fixation. Both patients recovered smoothly after the surgery.

**FIGURE 1:**
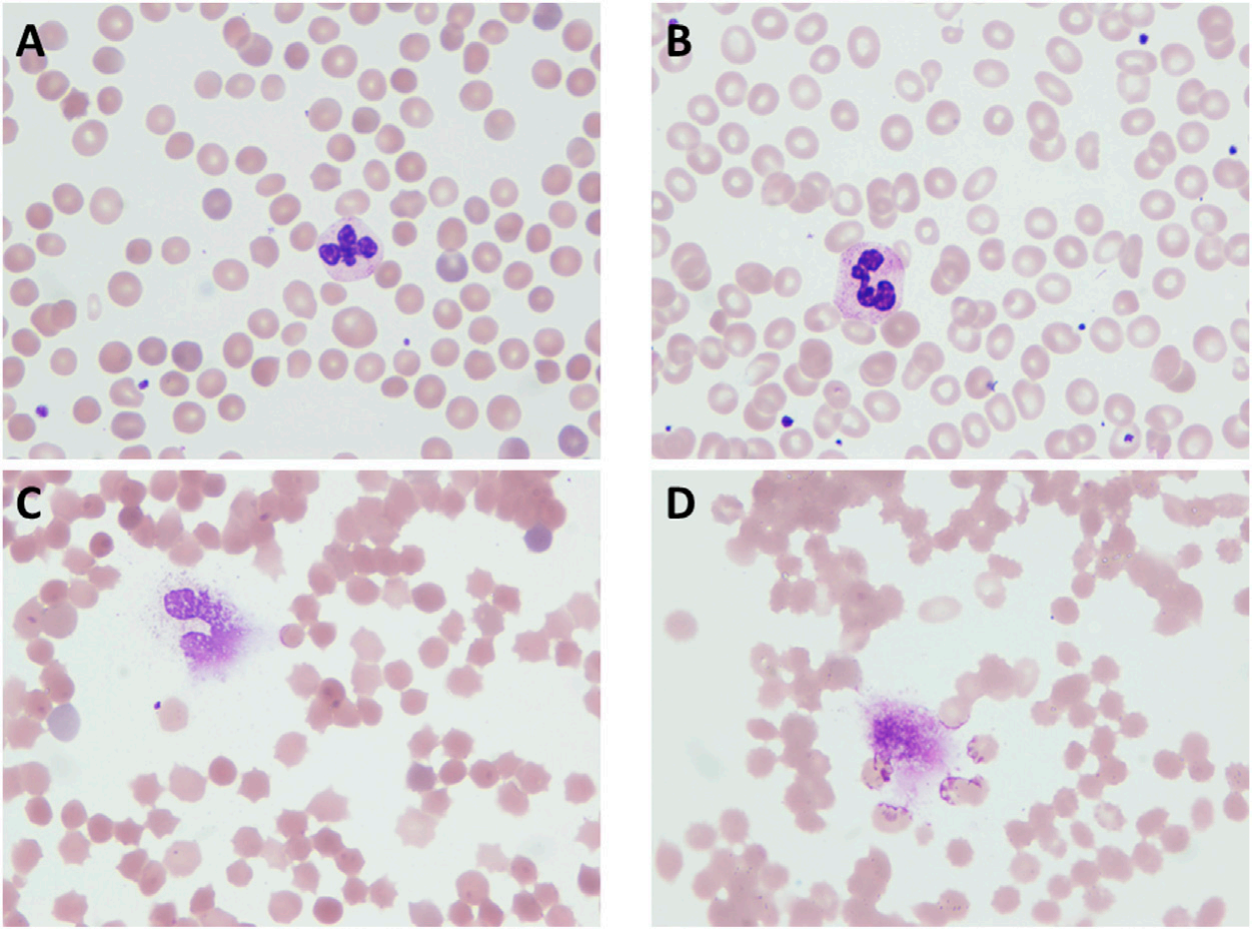
HS patient and normal erythrocytic patient blood cell smear slides. **(A)** Blood sample from HS patient’s peripheral vein before surgery; **(B)** blood sample from a normal erythrocytic patient’s peripheral vein before surgery; **(C)** blood sample from an HS patient’s Cell Saver salvaging system collection bag; **(D)** blood sample from a normal erythrocytic patient’s Cell Saver salvaging system collection bag.

Preoperative complete blood count (CBC, Siemens-Bayer, ADVIA 2120) of HS patient showed mild anemia (hemoglobin, 10.4 g/dl; hematocrit-Hct, 28.6%; platelets, 291 × 10^3^/μL; white blood cell, 10.1 × 10^3^/μL). Mild hemolysis was observed with elevated cell-free hemoglobin (cfHb 6.2 mg/dl, RIELE, Photometer 4040) and elevated bilirubin (TBil 95.1 μmol/L, DBil 8.9 μmol/L, Beckman Coulter, AU5821). The EOF test showed that the hemolysis started at saline concentration >0.6% NaCl and complete hemolysis at 0.36% NaCl. Preoperative CBC of the non-HS patient showed mild anemia (hemoglobin, 9.6 g/dl; hematocrit-Hct, 30.6%; platelets, 267 × 10^3^/μL; white blood cell, 5.99 × 10^3^/μL). No hemolysis was observed in the non-HS patient (cfHb 2.3 mg/dl, TBil 5.4 μmol/L, DBil 2.1 μmol/L), with the EOF test showing that the hemolysis started at a lower saline concentration of 0.48% NaCl and complete hemolysis at 0.28% NaCl. HS and non-HS patients’ blood smear slides indicated spherocytes and normal RBCs, respectively (Figures 1A,B).

Both patients received intraoperative autologous blood salvage with the same CS system (Cell Saver^®^ 5+, Haemonetics Corp, United States). During the 3-h surgery for HS patients, the amount of intraoperative bleeding was 400 ml, and 135 ml of RBCs was salvaged. During the 4-h surgery for non-HS patients, the amount of intraoperative bleeding was 500 ml, and 200 ml of RBCs was salvaged. The salvaged RBCs were infused into two patients, respectively. Blood smears showed that salvaged RBCs of either HS or non-HS patients appeared spiny after the processing of the CS system (Figures 1C,D). The EOF tests revealed mildly increased fragility in both HS RBCs (start hemolysis at >0.6% NaCl and complete hemolysis at 0.4% NaCl) and non-HS RBCs (start hemolysis at 0.52% NaCl and complete hemolysis at 0.28% NaCl). A drastic increase of cfHb was found in both HS blood savage (82.6 vs. 6.2 mg/dl) and non-HS blood salvage (57.9 vs. 2.3 mg/dl), as indicated by severe hemolysis during the CS process (Table 1).

**TABLE 1:** Comparative hemolysis-related test results of the HS patient and control patient.

	Control case		HS case		Normal range
	Pre-Op	Post-salvage	Pre-Op	Post-salvage	
FHb (mg/dL)	2.3	57.9	6.2	82.6	0–5
STH	0.48	0.52	>0.6	>0.6	0.48
CTH	0.28	0.28	0.36	0.4	0.28
EOF test	(-)	(-)	(+)	(+)	(-)

Pre-Op: blood sample drawn before the operation. Post-salvage: blood sample drawn from the collection bag after the autologous blood salvage. HS, hereditary spherocytosis; FHb, free hemoglobin; STH, the saline concentration that the hemolysis starts; CTH, the saline concentration that the hemolysis completes; EOF test, erythrocyte osmotic fragility test—a positive EOF test means a higher saline concentration to begin hemolysis compared with the normal range, no less than a 0.08% difference.

## Discussion

Up to now, hemolysis has been unavoidable during the CS process due to centrifuging, washing, and roller pumping of the RBCs (Valeri et al., 2006). For the packed or stored allogeneic RBCs, a mean cell-free hemoglobin concentration could increase from 72.6 mg/dl to 210.5 mg/dl after washing using the CS system. Despite the significantly increased post-wash cell-free hemoglobin, the washed allogeneic salvage was estimated to result in a mild and acceptable increase (3–5 mg/dl) in recipients’ cell-free hemoglobin (Welsby et al., 2021). For the autologous salvage in our report, much lower cell-free hemoglobin concentrations were found in either the HS (82.6 mg/dl) or normal erythrocytic (57.9 mg/dl) patient’s post-wash sample than in the washed allogeneic salvage, so neither patient manifested significant hemolysis after receiving the autologous salvage.

The spherical erythrocytes (spherocytes) are more fragile than normal erythrocytes, and research showed that stored spherocytes are more fragile and unsuitable for transfusion (Weinstein et al., 1997). The EOF test in our reports indicated that the CS process had no significant alteration on either patient’s erythrocyte fragility. However, being a report of only two patients, we did not perform isotope-traced RBC half-life measurements after the autologous transfusion. Both patients had a relatively small amount of blood loss and a small amount of salvaged RBC transfusion, so we cannot suggest a relatively safe amount of salvaged RBCs for transfusion under other surgical conditions for either HS or normal erythrocytic patients.

The Cell Saver^®^ (CS) system is designed for autologous blood salvage with the removal of broken blood cells and platelets, reduction of cytokines and chemokines, and increased hemolysis with an acceptable concentration of cell-free hemoglobin (Welsby et al., 2021). The quality of salvaged RBCs from the CS system after washing, centrifugation, and pump rolling, however, remains unclear, with the fragility of salvaged RBCs might be increased under some conditions (Chung et al., 2019), which could lead to more hemolysis after the salvaged RBCs are transfused back to the patient as osmotic fragility is an important determinant in the severity of hemolysis.

There have been reports on intraoperative cell salvage in other hemolytic anemia patients, mostly on sickle cell anemia (Black and Dearing, 1980; Brajtbord et al., 1989; Cook and Leland, 1990; Fox et al., 1994; Okunuga and Skelton, 2009; Firth and Szabo, 2010; You et al., 2017). Among the reported cases which performed blood smear after the salvage, more sickling was observed (Brajtbord et al., 1989; Cook and Leland, 1990; Okunuga and Skelton, 2009). Also, in one case (You et al., 2017), the patient suffered from hemolysis-induced renal failure after the transfusion of 300 ml salvaged RBC and 2U packed RBC. In one case (Brajtbord et al., 1989), the surgeons decided not to transfuse the salvaged blood due to a 50% incidence of sickling. While in other cases, patients recovered without transfusion-related adverse events. It is assumed that hypoxic atmosphere during the cell salvage process leads to more sickling in RBC from patients with sickle cell anemia, and a higher percentage of sickle hemoglobin (HbSS) is believed to be associated with more sickling. Thus, dilution of the salvaged RBC with more wash volume is suggested (Cook and Leland, 1990; Okunuga and Skelton, 2009). There are fewer reports on patients with other causes of hemolytic anemia receiving intraoperative cell salvage. To our knowledge, there are only two reports on patients with beta-thalassemia intermedia (Waters et al., 2003) and paroxysmal nocturnal hemoglobinuria (Kawamoto et al., 2019). Severe hemolysis was observed in both cases, and dilution of the salvaged blood appeared to be effective in the prior case. From the reported cases of intraoperative cell salvage for patients with hemolytic anemia, it appears that patients with hemolytic anemia are more prone to severe hemolysis during the cell salvage process. However, larger scale observation is needed to further exclude observation bias, and the underlying mechanism is in need of exploration.

From our report of two cases and previous reports, when considering the application of autologous blood transfusion for patients with hemolytic anemia, we should be aware that severe hemolysis could occur during the salvage process. Thus, we should evaluate the risk and benefits of autotransfusion based on the amount of salvaged blood and the patient’s needs and subsequently monitor for hemolysis-related complications in the patients.
